# PET Reveals Inflammation around Calcified *Taenia* *solium* Granulomas with Perilesional Edema

**DOI:** 10.1371/journal.pone.0074052

**Published:** 2013-09-13

**Authors:** Masahiro Fujita, Siddhartha Mahanty, Sami S. Zoghbi, Maria Desiree Ferraris Araneta, Jinsoo Hong, Victor W. Pike, Robert B. Innis, Theodore E. Nash

**Affiliations:** 1 Molecular Imaging Branch, National Institute of Mental Health, National Institutes of Health. Bethesda, Maryland, United States of America; 2 Laboratory of Parasitic Diseases, National Institute of Allergy and Infectious Diseases, National Institutes of Health, Bethesda, Maryland, United States of America; Institute of Neuroepidemiology and Tropical Neurology, France

## Abstract

**Objective:**

Neurocysticercosis, an infection with the larval form of the tapeworm, *Taenia* *solium* , is the cause of 29% of epilepsy in endemic regions. Epilepsy in this population is mostly associated with calcified granulomas; at the time of seizure recurrence 50% of those with calcifications demonstrate transient surrounding perilesional edema. Whether edema is consequence of the seizure, or a result of host inflammation directed against parasite antigens or other processes is unknown. To investigate whether perilesional edema is due to inflammation, we imaged a marker of neuroinflammation, translocater protein (TSPO), using positron emission tomography (PET) and the selective ligand ^11^C-PBR28.

**Methods:**

In nine patients with perilesional edema, degenerating cyst or both, PET findings were compared to the corresponding magnetic resonance images. Degenerating cysts were also studied because unlike perilesional edema, degenerating cysts are known to have inflammation. In three of the nine patients, changes in ^11^C-PBR28 binding were also studied over time. ^11^C-PBR28 binding was compared to the contralateral un-affected region.

**Results:**

^11^C-PBR28 binding increased by a mean of 13% in perilesional edema or degenerating cysts (P = 0·0005, n = 13 in nine patients). Among these 13 lesions, perilesional edema (n=10) showed a slightly smaller increase of 10% compared to the contralateral side (P = 0·005) than the three degenerating cysts. In five lesions with perilesional edema in which repeated measurements of ^11^C-PBR28 binding were done, increased binding lasted for 2-9 months.

**Conclusions:**

Increased TSPO in perilesional edema indicates an inflammatory etiology. The long duration of increased TSPO binding after resolution of the original perilesional edema and the pattern of periodic episodes is consistent with intermittent exacerbation from a continued baseline presence of low level inflammation. Novel anti-inflammatory measures may be useful in the prevention or treatment of seizures in this population.

## Introduction

Neurocysticercosis, due to infection with the cystic larval form of the tapeworm, *Taenia* *solium* , is the cause of about 29% of epilepsy in endemic regions [1]. Degenerating viable cysts commonly resolve into calcified cysticerci, which are found in 10-20% of individuals in randomly studied endemic populations and are commonly the foci of seizures and epilepsy [2]. Perilesional edema around calcifications is a recently described manifestation of neurocysticercosis. In a prospective series of 110 persons in Lima, Peru, with only calcifications due to neurocysticercosis and a recent history of seizures and a positive serology, 50% of those with recurrent seizures demonstrated perilesional edema [3]. The pathophysiology of perilesional edema is unclear but edema secondary to the seizure itself, release of ionized calcium, intermittent release of antigen from a calcified lesion and subsequent host inflammatory response or unknown causes are commonly suggested mechanisms [2,4].

Expression of translocator protein, (TSPO), located in the mitochondrial membrane is increased in inflammatory conditions of the brain [5]. TSPO is mostly up regulated in microglia, migrating macrophages as well as astrocytes [6,7]. Positron emission tomography (PET) scanning with a number of ligands has been used to quantitate TSPO expression [8]. We recently developed and validated the utility of a new ligand, ^11^C-PBR28 that has higher sensitivity to detect increases in TSPO compared to the first generation of ligand, ^11^C-PK 11195 [9,10,11].

To determine whether the perilesional edema is inflammatory in origin, patients followed prospectively under protocol for neurocysticercosis at the Clinical Center of the National Institutes of Health (protocol number 85-I-127) who presented with perilesional edema around calcifications or had degenerating *T* *. solium* cysticerci (cysts) were subjected to ^11^C-PBR28 based PET scanning.

## Methods

### Patients

The study was approved by NIH CNS Institutional Review Board, and written informed consent was obtained from all participants. Study subjects were recruited from a cohort of patients diagnosed with neurocysticercosis who were being followed prospectively as a part of an ongoing study of the natural history of neurocysticercosis (http://www.clinicaltrials.gov/ct2/show/NCT00526916?term=neurocysticercosis&rank=5). One hundred and nine patients were referred to NIH with the diagnosis of neurocysticercosis or cysticercosis for evaluation and/or treatment. All were able to travel to NIH, agreed to participate and signed a consent form. Of these, 58 are still being followed prospectively from 2 months to 28 years following enrollment. The patients studied had been followed under this protocol for 1-12 years before being enrolled in the PET scan protocol. Depending on the clinical situation, they were routinely evaluated by magnetic resonance imaging (MRI) every 6-12 months and/or when symptoms occurred. All had a history of seizures associated with the studied lesion(s), were taking anti-seizure medication with prior documented therapeutic drug levels.

Patients were studied under a number of situations (Table 1): 1) Following new symptoms and the demonstration of new or increased size of prior perilesional edema (new presence or increased size of the Fluid Attenuated Inversion Recovery (FLAIR)-positive signal on MRI); 2) After detection of unsuspected perilesional edema on routine MRI examination in asymptomatic patients; 3) Following a previous documented episode (1 month to 8 years earlier) of perilesional edema; and 4) Presence of a degenerating previously viable cyst. Degenerating cysts were studied because they are known to have inflammation. On the other hand, involvement of inflammation in perilesional edema was not known. In patients with multiple lesions, they could be studied for different purposes. For instance, in a newly diagnosed patient with perilesional edema both the current and previously involved second calcification could be analyzed. Perilesional edema was defined as the new appearance of FLAIR-high intensity that subsequently resolved. Gd-enhancement was also present around involved calcifications. Degenerating cysts were defined as a previously recognizable cyst that had decreased in size and lost its structure following anthelmintic treatment or after degeneration as a consequence of the natural evolution of disease. Surrounding Gd-enhancement was also present.

**Table 1:** Clinical Characteristics of Participants.

Patient #	Age	Sex	Serology for cysticercosis by Western blot	History	PET scan #	Days from new symptom or edema to PET scan
1	27	F	+	She had a long history of seizures with partial and secondary generalization. There were many calcifications on CT studies and MRI imaging documented multiple prior episodes of perilesional edema. A PET scan was performed after an episode of perilesional edema associated with left basal ganglion calcification causing right face tingling.	1	23
					2	63
2	24	F	+	The patient had a long-standing history of headaches and seizures. About 85 days prior to the PET scan she experienced generalized seizures. MRI showed two cysts, one in the right temporal cortex, provoking the seizure and another in the right frontal horn of the lateral ventricle. At the time of PET, there was a degenerating cyst in the temporal cortex.	1	85
3*	56	F	*+*	*The patient has a long history of seizures due to neurocysticercosis. Taeniasis was documented as a teenager in India followed by the onset of many seizure episodes due to untreated disease. After antihelminthic treatment in the mid 80s resulting in 55 calcified cysts, she experienced many documented episodes of perilesional edema and seizures or focal neurological symptoms. No perilesional edema episodes occurred for 8 years after treatment with methotrexate, a time when the first PET scan was done.*	*1*	*NA*
				Perilesional edema associated with dysphasia reoccurred after lowering the dose of methotrexate	2	13
				Another episode of perilesional edema associated with seizures occurred	3	13
*4*	*33*	*F*	*-*	*The patient complained of headaches, right leg numbness lasting for several days and focal seizures of the right leg and arm about 6 months before the PET scan. MRI showed perilesional edema around a large calcification in the left motor strip. Two other calcifications were also present in the brain parenchyma. At the time of the PET scan she was asymptomatic on anti-seizure medication and MRI showed no edema but enhancement around the calcification in the left motor strip.*	*1*	*NA*
5	19	M	+	The patient presented with generalized seizures caused by a degenerating enhancing edematous cyst in the right motor strip. No further seizures occurred after starting antiepileptic medication. The PET scan was performed 39 days after the seizure. The MRI showed enhancement without edema at the time of the PET scan.	1	39
6	40	M	-	The patient spent part of his childhood in Mexico where he was diagnosed with taeniasis. He was asymptomatic until 38 years of age, when he had a grand mal seizure associated with perilesional edema around one of >50 small calcifications. About 1 year later, he had another grand mal seizure with perilesional edema around the same calcification. He remained asymptomatic on anti-seizure medication. About 5 years later and 17 days before the PET scan, a routine MRI examination when the patient was asymptomatic, showed another episode of perilesional edema around the same calcification.	1	NA
7	42	M	-	The patient experienced a grand mal seizure associated with a single calcification with surrounding edema in the left motor strip 6 years prior to the PET scan. Anti-seizure medication was stopped about 5 months earlier. Twenty seven days before the PET scan, he experienced weakness on right arm and leg. MRI showed perilesional edema around the left motor strip calcification.	1	27
8	29	M	+	About a year prior to the PET scan, the patient presented with a right-sided weakness due to large edematous enhancing cyst in the left motor strip, which was surgically excised. He also had a viable cyst in the left frontal cortex, a large calcification in the left Sylvian fissure and a second small calcification in right parietal cortex; these calcifications had neither enhancement nor edema. The viable cyst responded to anthelmintic treatment. On withdrawal from steroids, both previously quiescent calcifications showed newly developed edema and enhancement associated with worsening headaches. The edema resolved without treatment. About 17 months later and 18 days prior to the PET scan, the patient experienced a grand mal seizure with massive edema around the left Sylvian fissure calcification.	1	18
9	30	F	+	The patient presented about 2 years prior to the PET scan with multicystic neurocysticercosis involving both ventricles and the parenchyma. The cysts in the ventricles caused hydrocephalus, which has been managed with shunt placement. The patient developed perilesional edema around a calcification in the left frontal cortex that also showed residual gliosis. The patient was asymptomatic at the time.	1	60

* This patient was the single American infected in India. Reported in [17,20]. All the other patients spend their childhood in heavily endemic counties of Central and South America.All the patients had certain or probable neurocysticercosis according to published criteria [21].Italics indicates the two cases that were studied remote from symptoms or edema.

### Data acquisition

PET data were obtained as described previously [10] after injection of ^11^C-PBR28 (Activity: 480 ± 89 MBq; Specific activity: 100 ± 60 GBq/µmol) and by acquiring data over the subsequent 2 h using the High Resolution Research Tomograph (HRRT; Siemens/CPS, Knoxville, TN, USA). One scan was performed using the Advance tomograph (GE Medical Systems, Waukesha, WI, USA) because the HRRT was not available. Metabolite-corrected arterial input function of ^11^C-PBR28 was measured in the first six scans but not in the subsequent six scans because the analysis of the first six scans showed that arterial data were not necessary to accurately measure the increase of ^11^C-PBR28 relative to the unaffected contralateral side. The ratios of total distribution volume, *V*_T_, measured with metabolite-corrected arterial input function and Logan plot [12] of the first six scans yielded virtually the same ratios of lesion to the contralateral side as the ratios of area under the curve (AUC) of brain activity (a difference of only 1·8 ± 1·0%, P = 0·86). In addition, in theory, the ratios of *V*_T_ should be essentially equivalent to the ratios of AUC because in the calculation of the ratios, arterial input data cancel out. Prior to PET scanning, none of the uninvolved, contralateral corresponding regions of the studied lesions demonstrated edema by FLAIR or Gd- enhancement for the previous one year.

At the time of each PET scan, an MRI scan was performed using 3 T Signa (GE, Milwaukee, WI) or 3 T Achieva (Philips Health Care, Andover, MA). The scan sequences included FLAIR and T1-weighted sequence with Gd enhancement to detect neurocysticercosis lesions and ~1 mm slice T-1 weighted volumetric images to co-register MRI to PET.

### Data analysis

Head motion during PET scans was corrected by monitoring its position during the scan with the Polaris Vicra Optical Tracking System (NDI, Waterloo, Ontario, Canada) [13] for HRRT or by realigning reconstructed images for the Advance tomograph. All MRI scans were co-registered to the volumetric image and then co-registered to the PET images.

All images were reoriented to a symmetrical position to draw a volume of interest (VOI) on the contralateral side of each lesion for comparison. VOI was drawn on MRI scans performed at the time of the PET scans; three Gd-enhanced lesions indicating degenerating cyst in three patients and 10 FLAIR-high intensity lesions indicating perilesional edema in eight scans of six patients (Table 2). One of the six patients (patient 3) had MRI abnormalities in different areas on two different occasions (scans 2 and 3). In addition, to assess the time course of the changes in ^11^C-PBR28 binding, two patients who showed perilesional edema 6 months and 8 years before the PET scan (Table 2, scan 1 of patient 3 and patient 4) were studied by drawing VOIs on these areas with old perilesional edema. To reduce noise, PET data obtained with HRRT were smoothed with full-width-half-maximum of 5 mm.

**Table 2:** Summary of PET findings.

					PET measurement of ratios to contra lateral side	
Patient #	Scan #	Type of lesion	Location	Size of lesion on MRI (cm^3^)	Area under curve	Logan
1	1	Perilesional edema	Lt. basal ganglia	2·56	1·14	1·14
		Perilesional edema	Rt. centrum semiovale	0·07	1·27	1·24
	2*	Perilesional edema	Lt. basal ganglia	2·56	1·11	1·12
		Perilesional edema	Rt. centrum semiovale	0·07	1·47	1·52
2	1	Degenerating cyst	Rt. temp. cx.	0·56	1·17	1·21
3	*1*	*Prior perilesional edema*	*Rt. Pari. cx.*	1·76	0·85	0·86
	2	Perilesional edema	Lt. temp. cx.	4·92	1·07	
		Perilesional edema	Rt. fron. cx.	0·07	1·11	
		Perilesional edema	Rt. occ. cx.	0·19	1·03	
	3**	Perilesional edema	Rt. temp. cx.	0·85	1·06	
*4*	*1*	*Prior perilesional edema*	*Lt. fron* *. cx.*	16·87	1·04	
5	1	Degenerating cyst	Rt. fron. cx.	0·19	1·32	1·29
6	1	Perilesional edema	Rt. temp. cx.	0·93	1·19	1·18
7	1	Perilesional edema	Lt. fron. cx.	0·90	1·08	
8	1	Degenerating cyst	Lt. pari. cx.	0·91	1·16	
		Perilesional edema	Lt. pari. cx.	2·83	1·18	
9	1	Perilesional edema	Lt. fron. cx.	4·08	0·90	
All active and new lesions			n = 13***		1·13±0·11	P=0·0005
Perilesional edema			n = 10		1·10±0·10	P=0·005

* The second PET scan of patient 1 was performed 40 days after the first PET scan. The same lesions were studied in the two PET scans by using the same regional drawing as done for scan 1.

** The third PET scan of patient 3 was done nine months after the second PET scan. Different lesions were studied in the two PET scans.

*** The second scan of patient 1 was not included because it studied the same lesions the first scan did.

Patient 3 was taking methotrexate at the time of scan 2.

Italics indicates old lesions.

Changes in ^11^C-PBR28 binding in lesions with MRI abnormalities were measured by calculating the ratios of the binding to the corresponding uninvolved region of the contralateral side, which showed no MRI changes except for the presence of small calcifications in some cases. The ratios were calculated in two ways: 1) using area under the curve (AUC) from time zero to 120 min. 2) in the first six scans in which metabolite-corrected arterial input function was measured, *V*_T_ was calculated in each voxel using Logan plot [12], and then the ratios to the contralateral side were calculated using *V*_T_. It is possible, but unlikely, that more than 1 year old inflammation on the contralateral side had prolonged increase in ^11^C-PBR28 binding (see Time course of the changes in ^11^C-PBR28 binding in Results).

Realignment and coregistration of images and smoothing were performed using Statistical Parametric Mapping (SPM) version 2005 (SPM 5) (Wellcome Trust Centre for Neuroimaging, London, UK. Volumes of interest and kinetic analyses were performed using PMOD 3.3 software (PMOD Technologies, Zurich, Switzerland).

### Statistical analysis

The increase of ^11^C-PBR28 binding in the lesions with MRI abnormalities was tested using one-tailed one-sample t-test. The difference in the ratios of ^11^C-PBR28 between lesion and the contralateral side by Logan plot and AUC was studied by two-tailed paired samples Wilcoxon Signed Rank test. Selection between parametric vs. non-parametric tests was determined based on Kolmogorov-Smirnov test for normality. p<0·05 was considered statistically significant. Results are shown as mean ± SD.

## Results

### Study group characteristics

The group studied consisted of 4 males and 5 females whose ages ranged from 19 to 56 years at the time of study, and who had been followed prospectively for 1-12 years (Table 1). The outline of histories of these patients is described below, and a detailed history for each patient is provided in Table 1. All except one (patient 3) were Hispanic from Latin America and had resided in *T* *. solium* endemic regions until they migrated to the United States. Seven of nine patients were included in this study because of a history of seizures associated with perilesional edema around the studied calcified cyst (patients 1, 3, 4, 6, 7, 8, 9). Of these, 6 were studied shortly after the perilesional edema event (patients 1, #3-scans #2 and #3, patients 6, 7, 8, 9) and 2 were studied at a time remote from the event (patient #3-scan #1, patient 4). Four of the 6 with current perilesional edema (patients 1, #3-scans #2 and #3, #7, and #8) presented with seizures or focal manifestations and the remaining patients (#6 and #9) were asymptomatic but were found to have perilesional edema at the time of a routine follow up MRI scan. Two subjects who had degenerating cysts (patients 2, 5), presented with seizures due to the studied lesion and underwent PET scans remote from the clinical event. Except for patient 9, evaluated calcified lesions demonstrated no perilesional edema in their previous most recent MRI performed because of symptoms or as part of the patient’s routine scheduled clinic visit. Because of a lapse of time before PET scanning could be arranged, six of the individuals (patients 1, 3, 6, 7, 8, and 9) still manifested perilesional edema at the time of the initial PET scans while in three (patients 2, 4, and 5) the edema had resolved. Patient 1 showed edema around more than one calcification, and patient 8 showed two lesions, perilesional edema associated with one calcification and gliosis around a second degenerated cyst (Table 2). Patient 1 had two PET scans about a month apart to determine the duration of increased TSPO uptake for a single episode of perilesional edema. Patient 3 who experienced multiple episodes of perilesional edema around calcifications since 1986 had three PET scans. This patient had been prescribed methotrexate 8 years earlier in an attempt to prevent perilesional edema and had not experienced any episodes before scan 1. She was initially studied while asymptomatic without perilesional edema (Table 1, scan 1) but subsequently developed two separate perilesional edema episodes. Two patients (#2 and #5) were studied (85 days and 39 days, respectively) after their initial presentation with seizures due to degenerating cysts. Patient 9 had multiple cysts both within the brain parenchyma and ventricular system. The latter caused hydrocephalus and shunts were placed 19 months before the PET scan. This patient experienced a seizure associated with perilesional edema around a calcified lesion in the left frontal cortex 17·5 months before the PET scan. The edema indicated by FLAIR-high intensity region almost resolved after 2·5 months but a new FLAIR-high signal in the same area re-occurred 15·5 months after the seizure and persisted for approximately 2 months before the PET scan. The increased size of the FLAIR-high signal region was consistent with worsening of perilesional edema, and prompted the PET scan. However, the patient was asymptomatic at the time of the PET scan. The persisting FLAIR signal for two months before PET is consistent with the presence of gliosis in addition to edema since gliosis is a non-resolving lesion that demonstrates a FLAIR signal without enhancement. PET measurement in patient 9 was done only for the perilesional edema in left frontal cortex; other lesions were not suitable for measurements because most were small and a sizable part of the lesion was calcified.

### PET and blood data

**Figure 1:**
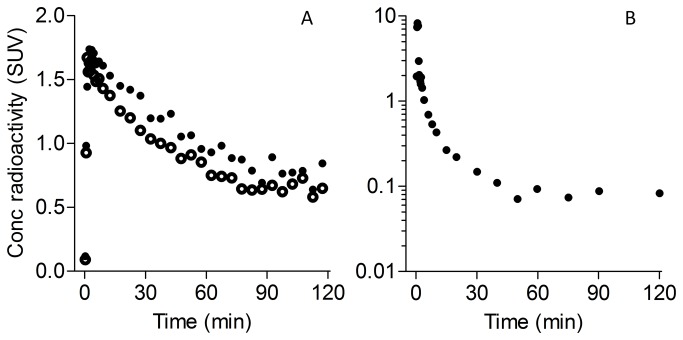
Positron emission tomography measurement of ^11^C-PBR28 binding. In the first six scans including scan 1 of patient 1 shown in Figure 1, ^11^C-PBR28 binding was measured as total distribution volume, *V*_T_, using Logan plot [12] based on radioactivity measured by the PET scanner (A, closed circles: perilesional edema in left basal ganglia, open circles: contralateral side with normal MRI) and ^11^C-PBR28 concentration in arterial plasma measured with radio-high performance liquid chromatography (B). Brain activity decreased to half of the peak in about 60 min indicating that none of the participants including this patient was a low affinity binder [14] to ^11^C-PBR28. Both the analyses using only area under the curve of brain radioactivity (A) and that using both brain (A) and arterial blood data (B) gave the same increase of 14% in ^11^C-PBR28 binding indicating that arterial blood sampling was unnecessary.

Based on washout of the brain PET data, all participants were high- or mixed-affinity binders for PBR28 for whom the binding to TSPO is measurable, and none was a low affinity binder for whom the binding is not measurable [14]. After injection of ^11^C-PBR28, brain activity peaked at ~2 SUV and then decreased in a similar way as we observed in previous studies using high- and mixed-affinity binders of PBR28 (Figure 1-A). ^11^C-PBR28 peaked at ~12-20 SUV in arterial plasma and decreased quickly as we previously observed in high- and mixed-affinity binders (Figure 1-B).

### ^11^C-PBR28 binding in areas with new MRI abnormalities

**Figure 2:**
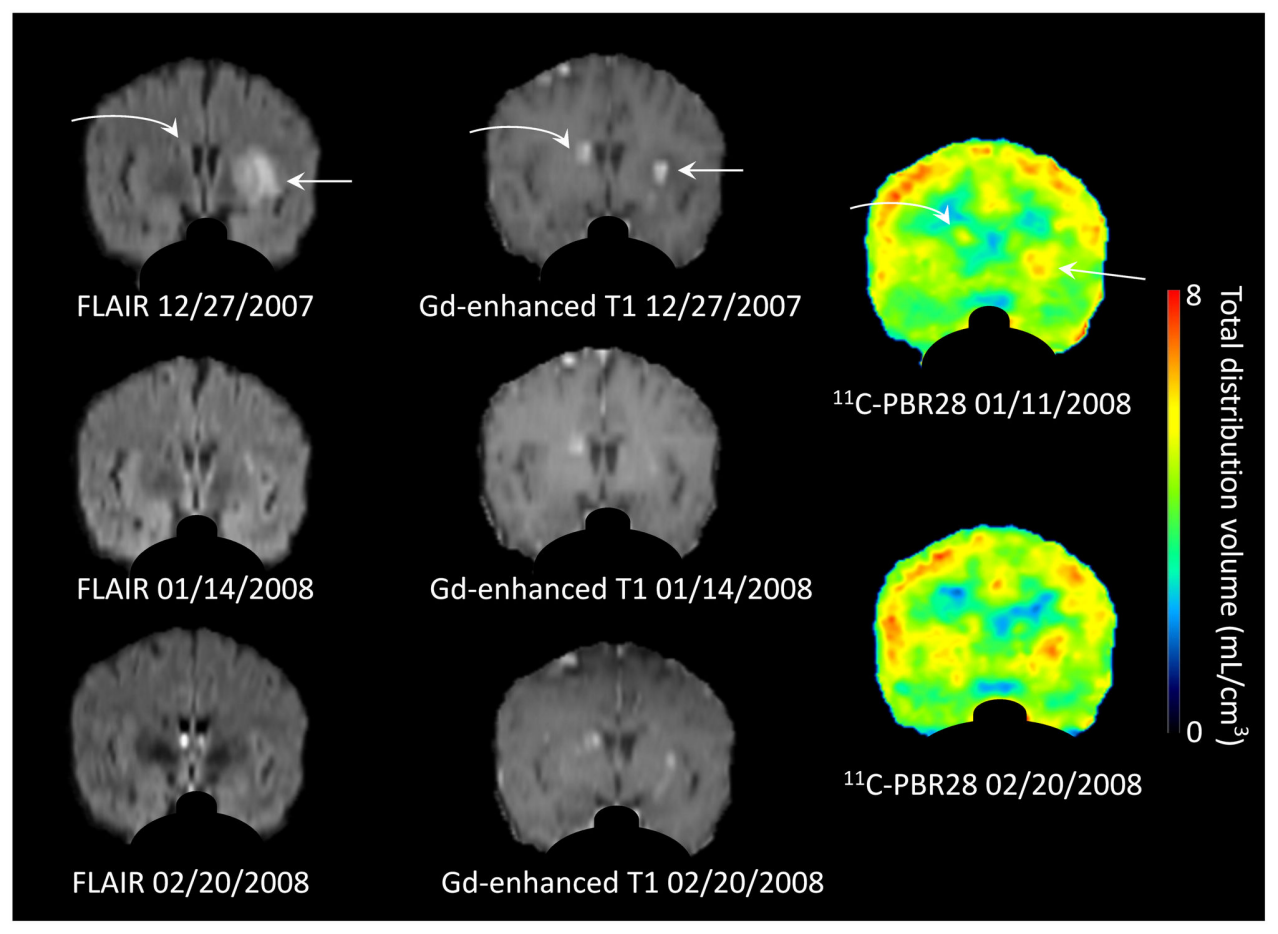
MR and PET images of patient 1 following a single perilesional edema episode. Fluid-attenuated inversion recovery (FLAIR) and gadolinium (Gd)-enhanced T1-weighted MR images at three time points and ^11^C-PBR28 PET images obtained at two time points. Two lesions are seen, the straight arrow points out a calcification with edema in the left basal ganglion and the curved arrow the edema and calcification in the right centrum semiovale. Although perilesional edema resolved by 01/14/2008, ^11^C-PBR28 PET uptake was still present. PET images show ^11^C-PBR28 binding in total distribution volume, *V*_T_. The images are reoriented to show the two lesions on the same coronal slice. Images outside of brain parenchyma are masked.

**Figure 3:**
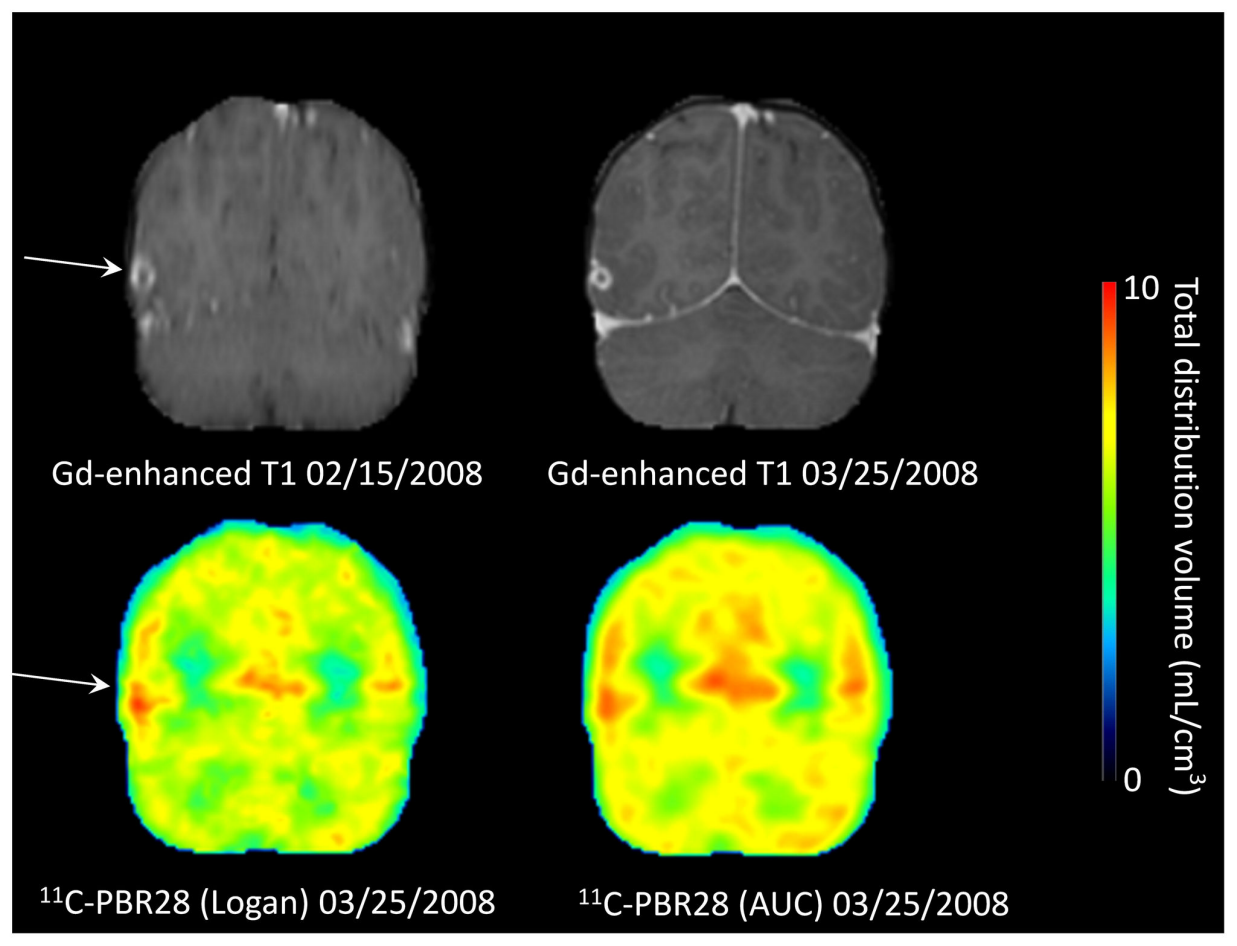
MR and PET images of patient 2 who showed a degenerating cyst (arrow). The patient presented with a seizure 85 days (01/06/2008) before the PET scan on 03/25/2008. On 02/15/2008 when the patient was asymptomatic, MRI scan showed a Gd-enhanced degenerating cyst in right temporal cortex (arrow). On the day of the PET scan (03/25/2008), the Gd-enhanced lesion slightly shrunk, and the PET scan showed increased ^11^C-PBR28 binding based on Logan plot (21%) and area under the curve (AUC, 17%). The color bar applies to only the PET image on the left side.

All but one lesion (patient 9), including both perilesional edema and degenerating cysts (n = 13 in Table 2 not including old lesions indicated by Italics), showed an increase in ^11^C-PBR28 binding based on AUC analysis on initial evaluation (excluding repeated measurements of the same lesions). The average increase of 13% (ratio to the contralateral side = 1·13) was highly significantly (p = 0·0005). The 10 perilesional edema lesions showed a significant increase of 10% (ratio to the contralateral side = 1·10, p = 0·005, a case with perilesional edema is shown in Figure 2) while the three degenerating cysts in patients 2, 5, and 8 tended to show somewhat greater binding values of 17%, 32%, and 16% (a case with degenerating cyst is shown in Figure 3).

### Time course of the changes in ^11^C-PBR28 binding

In addition to studying changes in ^11^C-PBR28 binding within 2 months of perilesional edema episodes, we studied patients at later time points, up to 8 years (Table 3). Prolonged increase in ^11^C-PBR28 binding was found in five lesions 2-9 months after the development of new symptoms (right centrum semiovale and left basal ganglion of patient 1 and left temporal, right frontal, and right occipital cortices in scans 2 and 3 of patient 3). The values of ^11^C-PBR28 binding in 3 of the 5 (scan 2 compared with #1 of patient 1 and scan 3 compared with #2 of patient 3) were increased above the scans obtained earlier, a few weeks after the single perilesional edema episode. On the other hand, no increase in ^11^C-PBR28 binding was detected 0·5-8 years following the last perilesional edema episode (scan 1 of patient 3 and scan 1 of patient 4). Patient 1 is illustrative (Figure 2). This patient experienced tingling of the right face and arm as well as new perilesional edema around a calcification in the left basal ganglia (Figure 2, straight arrow) and also slight edema in right centrum semiovale (Figure 2, curved arrow) documented by MRI on 12/27/2007. Repeat MRI on 01/14/2008 revealed markedly decreased edema in both lesions but the first PET scan on the same date still showed 14% and 27% increase in ^11^C-PBR28 binding in left basal ganglia and right centrum semiovale, respectively. Repeat MRI and PET scans on 02/20/2008 showed that edema had almost disappeared but Gd-enhancement persisted. ^11^C-PBR28 binding showed increases of 11% and 47% in left basal ganglia and right centrum semiovale, respectively. Therefore, perilesional edema disappeared by day 63 after the onset of symptoms (02/20/2008) but the increase of ^11^C-PBR28 binding persisted. Thus, it appears that an increase of binding persists in some lesions up to 9 months after a single episode and may persist even longer. The patient studied 8 years following many episodes of perilesional edema demonstrated no increase of uptake suggesting eventual normalization. However, this patient was taking methotrexate, which could have decreased uptake.

**Table 3:** Time course of the changes in ^11^C-PBR28 binding in patients who showed perilesional edema.

Patient #	Location	Time after symptoms and MRI changes	PET ratio to contralateral by AUC	Time after symptoms and MRI changes	PET ratio to contralateral by AUC
1		Scan 1		Scan 2	
	Lt. basal ganglia	23 days	1·14	63 days	1·11
	Rt. centrum semiovale		1·27		1·47
3		Scan 1			
	Rt. pari. cx.	8 years	0·85		
		Scan 2		Scan 3*	
	Lt. temp. cx.	13 days	1·07	282 days	1·10
	Rt. fron. cx.		1·11		1·04
	Rt. occ. cx.		1·03		1·22
4	Lt. fron. cx.	6 months	1·04		

Changes in ^11^C-PBR28 binding in newly detected perilesional edema were studied at two time points in scans 1 and 2 of patient 1 and scans 2 and 3 in patient 3. Possible prolonged changes in ^11^C-PBR28 binding were studied in areas with prior perilesional edema in scan 1 of patient 3 and in the scan of patient 4.

* To study the time course of the changes, ^11^C-PBR28 binding in the lesions detected in scan 2 was measured again in scan 3. Therefore, none of these lesions is the same as the lesion reported in patient 3 scan 3 in Table 2.

AUC: area under the curve.

## Discussion

The increase of TSPO detected in the current PET study indicates the presence of activated microglia, reactive astrocytes and macrophages in perilesional edema lesions. The interpretation of the PET findings is consistent with an inflammatory etiology of the perilesional edema and is supported by direct histopathological examination of 2 surgically removed calcified cysts associated with perilesional edema and seizures ( [15], Houpt et al. in preparation). These lesions demonstrated significant inflammation with infiltration of macrophages, microgliosis and activated astrocytes [15], (Houpt et al. in preparation). Additionally, the presence of Gd-enhancement around some but not all calcified granulomas indicative of blood brain barrier dysfunction suggests the existence of sustained inflammation around calcified granulomas [16]. It is well established that there is a significant host inflammatory response to degenerating cysts, which sometimes cause episodic seizures and newly appreciated perilesional edema around the degenerating cyst. These cysts may or may not calcify. The most reasonable hypothesis is that in the process of the progression from degenerating to calcified cysts, some calcified cysts, unlike most, continue to maintain some degree of inflammation. Similar to degenerating cysts, for unclear reasons, calcified cysts episodically invoke an enhanced inflammatory host response, which manifests as perilesional edema around the calcified cyst. Although the present study does not allow us to definitively conclude whether the inflammatory response, visualized as perilesional edema, is the cause of seizures, if the above hypothesis is correct, then the inflammatory response is the primary cause of the seizures.

From the above the persistence of TSPO increase after a single event suggests the presence of low level, long-term ongoing inflammation and may explain why only certain calcifications demonstrate perilesional edema and why these are commonly involved in repeated perilesional edema episodes. Only one patient (#9) showed a decrease in ^11^C-PBR28 binding with the presence of perilesional edema. As noted above, the lesion differs because unlike all the other lesions studied, it demonstrated an increased signal in FLAIR before the edema again increased.

At the time of the PET scans, all patients were taking anti-seizure medications. In addition, patient 3 was orally taking methotrexate to suppress excessive host immune reactions [17]. No patient was taking corticosteroids. Anti-seizure medications are unlikely to have affected the results of the PET scans because, to our knowledge, there is no publication reporting the influence of such medications on neuroinflammation or TSPO. On the other hand, methotrexate might have suppressed an increase in activated microglia and reactive astrocytes and also the increase in TSPO because methotrexate has been used to suppress excessive inflammatory reactions in neurocysticercosis [17] and other disorders such as rheumatoid arthritis [18].

The results reported above and additional analyses confirmed that the increase of ^11^C-PBR28 binding was caused by increase in TSPO but not by artifacts. Because inflammation may change permeability of chemicals through the blood brain barrier, there may be suspicion that the PET measurement in this study was influenced by the changes in blood brain barrier. This is unlikely because we measured both uptake to and washout from brain over two hours. In addition, the theory of pharmacokinetic analyses states that the measurement using arterial-input function corrects influences from the changes in permeability [19]. The measurement of ^11^C-PBR28 binding using metabolite-corrected arterial input done in the first half of the scans of the current study showed results almost identical to those by only brain data. Another possible concern is the influence of radiometabolites of ^11^C-PBR28 to the PET measurement. Because the ratios of radiometabolites / ^11^C-PBR28 increase over time in arterial plasma [10], PET data at later time points may be more influenced by accumulating radiometabolites. Additional analysis showed this possibility is very unlikely because calculations using the 90 min PET data, a time when metabolites have not accumulated as much compared to the 120 min, yielded almost the same results, only 2·2% smaller ratios to the contralateral side than that obtained from 120 min data.

In conclusion, increased uptake of ^11^C-PBR28 was present in patients with acute perilesional edema events. Persistence of increased uptake continued for undefined longer duration of time, which is likely indicative of low grade continued inflammation. Although it is not possible to determine direct causation based on the results of this study, integrating knowledge of the pathophysiology of inflammation in neurocysticercosis with the finding that perilesional edema is inflammatory in origin, we suggest inflammation is the primary mechanism of seizure activation in perilesional edema associated with calcifications and therefore increased ^11^C-PBR28 uptake around specific calcifications may indicate a heightened risk for seizures provoked at these foci. We suggest that perilesional edema episodes are due to the persistence of low grade inflammation driven by the presence, intermittent recognition of residual *T* *. solium* antigens or periodic loss of suppression of the inflammation at the calcified focus. Measures to inhibit inflammation in addition to anti-seizure treatment might be helpful in control of epilepsy due to this phenomenon. ^11^C-PBR28 PET can be a useful measure of the effectiveness of the medication.
